# Effects of a School-Based Behavior Modification Program on Body Composition, Dietary Behavior, and Physical Activity in Overweight High School Students in Rural Northern Thailand: A Randomized Controlled Trial

**DOI:** 10.34172/jrhs.2025.179

**Published:** 2025-04-01

**Authors:** Anavin Phattharaphakinworakun, Thidarat Somdee, Supattarayan Thongjit, Suneerat Yangyuen

**Affiliations:** ^1^Faculty of Public Health, Mahasarakham University, Thailand; ^2^Institute of Dentistry, Suranaree University of Technology, Thailand

**Keywords:** Physical activity, Food consumption, Behavior modification, High school students, Overweight

## Abstract

**Background:** Behavioral modification programs have improved body composition, dietary behavior (DB), and physical activity (PA). However, there is limited evidence on the effectiveness of these programs among overweight high school students in rural areas of Thailand. Therefore, this study aimed to examine the effects of a school-based behavioral modification (SBM) program on these factors among high school students with overweight.

**Study Design:** This study employed a randomized controlled trial.

**Methods:** The study was conducted from November 2022 to May 2023 among overweight high school students. A total of 100 overweight students were randomly assigned to either an intervention (n=50) or a control (n=50) group. The intervention group received an SBM, while the control group received the usual educational program. The chi-square test, independent-samples t-test, Mann–Whitney U test, ANOVA, and Cochran’s Q test were used to analyze data.

**Results:** Both groups were female (72.0%), with a mean age of 17.03 years. At the follow-up, the intervention group demonstrated significant improvements in DB and PA and a reduction in sedentary behavior compared to the control group (*P*<0.05). Additionally, statistically significant differences were observed between the intervention and control groups regarding biceps (*P*=0.001), triceps (*P*=0.031), and waist circumference (*P*<0.001).

**Conclusion:** The SBM effectively increased students’ PA, improved DBs, decreased sedentary behavior, and resulted in changes in body composition. These findings indicated that SBM programs are useful for healthcare providers or teachers to promote healthy behaviors among students and can be applied in related research in different contexts and situations.

## Background

 Being overweight among high school students represents significant global health concerns with, approximately 1 in 5 experiencing excess weight.^1^ The prevalence of overweight was 18.0% in 2020 and is expected to increase to 38.0% by 2035.^2^ Approximately 41.3% of people at high risk for overweight experience health problems related to weight, both physical and psychological. ^3,4^ especially contributing to non-communicable diseases (NCDs). Approximately 92 million people worldwide are projected to have premature mortality due to these issues by 2050.^5^

 Previous studies have revealed that a behavioral modification (BM) program is a tool designed to change body composition, weight, and lifestyle habits among obese or overweight individuals.^6^ Most BM programs focus on increasing physical activity (PA) levels, decreasing sources of sedentary lifestyles, and improving dietary behavior (DB).^6,7^ Additionally, BMs targeting diet and PA changes are the cornerstones of interventions for weight management in overweight and obese populations and are effective in reducing weight and improving health, at least in the short term.^7^ Effective BM methods include self-monitoring, cognitive-behavioral approaches, and problem-solving.^6^ A literature review showed that school-based behavioral modification (SBM) programs are effective in treating and preventing overweight and obesity in adolescents. Examples of these programs include classroom lessons, education programs focused on increasing PA and discouraging sedentary lifestyles, and programs promoting a healthy diet.^8,9^ Such SBMs have led to increased PA, improved DB, and changes in triceps skinfold thickness, and body mass index (BMI) Z-scores among overweight or obese adolescents.^8,10^ Moreover, BM interventions are more effective when implemented at a younger age. Schools are recognized as health-promoting environments, as adolescents spend a significant part of their day at school.^11^ Thus, applying SBMs to improve health behaviors and body composition may benefit overweight students.

 In Thailand, 35.2% of high school students in rural areas of Northern Thailand are overweight.^12,13^ According to the Thai Health Report in 2022, poor eating habits are detrimental to health, with an increase in the consumption of sweetened beverages, processed meats, and semi-prepared foods and high-fat foods (17.3%, 22.9%, and 26.3%, respectively). Additionally, 16.1% of students reported engaging in adequate PA, while 69.5% reported sedentary behavior.^12,14^ Thus, high school students may be at risk of being overweight, potentially leading to obesity diagnoses and the prevalence of NCDs.^13,14^ BM programs have been used to modify health behavior among overweight and obese students, most of which focus on family- and community-based interventions.^15^ However, school-based programs remain limited, primarily focusing on nutritional interventions, and few studies have combined BM techniques addressing both diet and PA.^16^ Therefore, this study aimed to examine the effects of an SBM program on health behavior and body composition among overweight Thai high school students, which may help promote healthier behaviors.

## Methods

###  Study design and setting

 This randomized controlled trial was conducted from November 2022 to May 2023 among overweight high school students in a rural area in Mae Hong Son Province, Northern Thailand.

###  Study participants

 The sample size was calculated using the formula:

$n/group=\left(Z_{1-\alpha /2}+Z_{1-\beta }\right)2\;\left[\sigma_{trt}^{2}+\left(\sigma_{con}^{2}/r\right)\right]/{\left(\mu_{con}-\mu_{trt}\right)}^{2}$ . ^17^

 This calculation was based on a study by Duangchan et al,^18^ considering a mean difference (µ_con_* - *µ_trt_) of 0.35 in average BMI before and after the intervention, with a standard deviation (SD) of body weight in kilograms $\left(\sigma_{trt}^{2}\right)=2.31$ in the experimental group and (*σ*_con_) = 3.75 in the control group. The experiment: control ratio (r) was set at 1, with a 95% confidence interval, a 90% test power, and a 10% dropout rate. The sample size calculation was as follows:

 n/group = (1.58) [5.337 + 14.063] / (0.35) ^2^ = (1.58) (19.40) / 0.7 = 30.7 / 0.7 = 43.86 ≈ 44

 Hence, with a dropout rate of approximately 10%., the total sample size required for each group was 50, and the total sample size was 100. Simple random sampling was used to select 16-17 students per grade from three grade levels for the intervention and control groups. The inclusion criteria were high school students studying in the target schools, overweight students classified with a BMI for their age greater than or equal to the 85th percentile or ≥ + 1 SD.^19^ students’ consent to participate in the study, parental permission for their children to participate in the study, and no physical or mental illness.^5^ Exclusion criteria included unwillingness to continue participation, absence from more than three intervention sessions, incomplete questionnaires, and dropout during the study.

###  Recruitment and randomization 

 The recruitment and randomization of participants were performed by a researcher who was not involved in the intervention or outcome assessment. The rural high schools in Mae Hong Son province were divided into north and south clusters based on geographical location. Two high schools were then selected from the seven resulting clusters and randomly allocated in a 1:1 ratio to either the control group (south part) or the intervention group (north part) using the cluster sampling method. Then, students within each selected school who met the inclusion criteria were chosen using computer-generated random numbers. Each school consisted of three grade levels, and simple random sampling was used to select 16-17 students per grade for the control and intervention groups. Thus, 100 students entered the study and were randomly assigned to the intervention and control groups, with 50 students in each group.

###  Data collection tools and techniques 

 Socio-demographic variables included age, sex, weight, height, and BMI for age. The digital weighing scale was operational and adequately powered. A calibration test was performed to ensure functionality. Thick clothing, including shoes and socks, was removed to minimize excess weight. Participants were required to stand still and upright on the scale for accurate measurement. Weight was recorded with a precision of 0.1 kg, and participants were weighed while fasting, preferably at 15:00 hours. Height was measured using a stadiometer with a width of no less than 5 cm. Measurements were taken in the standard posture, that is, standing erect with the head, back, legs, and heels against the wall. Height was recorded to the nearest 0.1 cm.^20^ BMI-for-age was calculated using weight (kg) and height (cm) based on the percentile standards developed by the U.S. Centers for Disease Control and Prevention (CDC) for children and adolescents aged 5–19 years. An overweight range was defined as the 85th to less than the 95th percentile, or from + 1 SD to + 2 SD.^19^

 Body composition evaluation comprised the assessment of subcutaneous fat accumulation, which was assessed using skinfold caliper measurements at four positions, with results expressed in millimeters (mm). Measurements were taken at subcutaneous locations to determine body fat percentage by age and gender. Calculations were based on the equation developed by Durnin and Womersley.^21^ The four skinfold thickness (in mm) were measured as follows:

1. Biceps: Subcutaneous fat at the midpoint of the anterior upper arm muscle (mm);
2. Triceps: Subcutaneous fat at the midpoint of the posterior upper arm muscle (mm);
3. Suprailiac: Subcutaneous fat above the iliac crest, measured diagonally higher than the iliac crest (mm);
4. Suprailiac: Subcutaneous fat below the scapula (mm). ^21^

Measurements were conducted three times at each location to estimate the average of the positions among male and female participants.

 Body fat (mm) was calculated using Durnin and Womersley equations:

 Body fat = (495/D) – 450

 where D = body density, L = log (sum of four skinfolds; biceps, triceps, subscapular, and suprailiac).

 For the age group < 17 years, body density was calculated as:

 D = 1.1533 - (0.0643 L) for boys

 D = 1.1369 - (0.0598 L) for girls

 For the age group 17–19 years, the formula was:

 D = 1.1620 - (0.0630L) for boys

 D = 1.1549 - (0.0678L) for girls.^21^

 Waist circumference (cm) was assessed using a standard measuring tape at the midpoint between the lower between the lower rib margin and the top of the iliac crest (cm).^22^

 Hip circumference (cm) was assessed using a standard measuring tape to measure the circumference of the external pelvic area (cm) at the pelvic position.^22^

 DB was measured using the Thai Recommended Daily Intake scale (Thai RDI).^23^ consisting 36 items with a total score of 216. The DB scores were categorized into three levels^23^: good eating habits (157–216 points), moderate eating habits (97–156 points), and poor eating habits (36–96 points). Cronbach’s α of 0.96 indicates good internal consistency.

 PA and sedentary behaviors were assessed using the Thailand PA Children Survey scale for recording the PA over the past 7 days, including weekdays and weekends. It comprises 13 questions recording activity patterns and durations (in minutes) per day.^24^ Cronbach’s α = 0.74 indicates that the scale has good internal consistency. The PA and sedentary behaviors were categorized as follows:

- Adequate PA: This involves an average of 60 minutes of moderate-to-vigorous PA across the week, including aerobic exercises, walking, running, swimming, and sports activities. Vigorous-intensity aerobic activities and muscle-strengthening exercises should be incorporated at least 3 days a week.^2,25^
- Prolonged sedentary behavior: Respondents were asked about time spent in sedentary activities per day (e.g., watching television, playing computer/video games, and sitting and reading). Respondents were considered to have prolonged sedentary behavior if they spent more than 2 hours per day on such activities.^2,25^

###  Intervention

 After a literature review and discussion with specialists in health education, health behavior, and adolescent psychology, a school-based intervention based on social cognitive theory was developed. The SBM focused on dietandPA, consisting eight sessions with a total of eight activities, each weekly session lasted 80 minutes. The intervention was delivered to the intervention group over eight weeks, followed by a four-week follow-up period (Table 1). Each session was structured as follows:

**Table 1:** Summary of the school-based behavioral modification program

**Session**	**Topics**	**Objectives**
1	Snack and dessert consumption	To introduce participants to the nutritional data and appropriate proportions for snack and dessert consumption
2	Fast-food and condiment consumption	To inform participants about how to reduce fast-food consumption and the recommendation of daily intake amounts of condiment usage
3	Unhealthy consumption behaviors	To introduce participants to the risk of NCDs and complications from unhealthy consumption behaviors
4	Goals setting for behavior change	To assist participants in identifying specific behaviors to change and how to go about doing so.
5	Food records	To show participants how to record food consumption daily.
6	Body composition assessment	To show participants how to assess body fat and BMI for age by using anthropometric equipment
7	Evaluating physical activity and sedentary behavior	To demonstrate participants how to monitor and evaluate physical activity and sedentary behavior and promote more physical activity
8	Experiences of behavior change	To motivate participants to share with group members their experiences of how to change in dietary and physical activities

*Note.* BMI: Body mass index; NCD: Non-communicable disease.

####  Session 1: Snack and dessert consumption

 Participants learned about the appropriate portion sizes for snacks and desserts, how to read nutritional labels, and how to utilize media from convenience store snack packages.

####  Session 2: Fast-food and condiment consumption

 Participants learned about the appropriate portions for fast food and condiment usage, illustrated using realistic food images. The recommended daily intake, measured in tablespoons, was emphasized.

####  Session 3: Unhealthy consumption behaviors

 Participants were instructed on NCDs and complications arising from unhealthy consumption behaviors, using video media and lifelike disease illustrations.

####  Session 4: Goal-setting for behavior change

 Participants practiced setting personal goals for behavior change, focusing on increasing PA and healthy DB and reducing sedentary behavior.

####  Session 5: Food records

 Participants practiced how to keep food records using a diary to document their main meals (three meals) and snack times.

####  Session 6: Body composition assessment

 Participants were taught how to assess body fat and BMI for age using skinfold calipers, circumference tape, weighing scales, and height measurements.

####  Session 7: Evaluating PA and sedentary behavior

 Participants practiced self-monitoring and evaluating their PA and sedentary behaviors. Additionally, they learned to calculate the time spent on physical activities and sedentary behavior using electronic media and realistic illustrations.

####  Session 8: Experiences of behavior change

 Participants were encouraged to share their experiences of behavior change related to dietary habits and PA with their group members.

 During the follow-up period, a 10-15 minute group discussion was conducted once a week for three weeks, which focused on behavior change, troubleshooting challenges, and keeping motivation to continue the effects of the intervention. At the end of week four, a school visit was made for outcome measurements.

###  Control group

 Participants in the control group did not receive any components of the SBM program. Instead, they continued with their regular health education classes, which were offered by the teacher.

###  Study procedure

 Both groups underwent baseline assessments, with the intervention group receiving SBM, while the control group did not. Outcomes were concealed and measured after the intervention and at follow-up using the same data collection methods.

###  Data analysis

 Descriptive statistics were used to analyze the participants’ characteristics and outcomes. Chi-square tests were used for categorical variables, independent-sample t-tests were used to continuously distribute variables with a normal distribution, and the Mann–Whitney U test was used for skewed continuous variables to compare the baseline characteristics of participants between the intervention and control groups. Outcome measurements were compared before intervention, after intervention completion, and at follow-up visits. For within-group comparisons, repeated-measures ANOVA was used to assess changes in normally distributed continuous variables, while Cochran’s Q test was employed for categorical variables. For comparison between the intervention and control groups, independent-sample t-tests and chi-square tests were used for changes in normally distributed continuous variables and categorical variables, respectively. All statistical analyses were carried out using SPSS version 20.0 (IBM Corp., Armonk, NY, USA), with a *P* value < 0.05 considered statistically significant.

## Results

 A total of 109 students were assessed for eligibility, of which, nine students were excluded, leaving 100 participants who were enrolled at baseline and completed the 4-week follow-up. Most participants were female (72.0%), with a mean age of 17.03 years (SD = 1.71). Approximately, 51.0% had poor eating habits, 73.0% reported engaging in PA, and 96.0% reported prolonged sedentary behavior. The baseline mean (SD) for body composition parameters were as follows: biceps = 12.64 (4.16), triceps = 13.42 (4.41), subscapular = 23.34 (4.38), suprailiac = 21.39 (5.17), sum of four skinfold thicknesses = 70.79 (12.38), body fat = 29.50 (3.74), waist circumference = 86.80 (10.81), hip circumference = 99.40 (9.88), weight = 76.90 (12.94), height = 165.01 (7.83), and BMI for age (kg/m^2^) = 28.14 (3.59). The baseline characteristics showed no statistically significant differences between the intervention and control groups (Table 2).

**Table 2:** Baseline characteristics of participants

**Characteristics**	**Total (N=100)**		**Intervention (n=50)**		**Control (n=50)**		***P*** ** value**
	**Number**	**Percent**	**Number**	**Percent**	**Number**	**Percent**	
Categorical variables							
Sex							0.910
Female	72	72.0	38	76.0	34	68.0	
Male	28	28.0	12	24.0	16	32.0	
Dietary behavior							0.137
Good eating habits	26	26.0	16	32.0	10	20.0	
Moderate eating habits	23	23.0	10	20.0	13	26.0	
Poor eating habits	51	51.0	24	48.0	27	54.0	
Physical activities							
Adequate physical activity	73	73.0	38	76.0	35	70.0	0.312
Prolonged sedentary behavior	96	96.0	50	100.0	46	92.0	0.425
	**Mean**	**SD**	**Mean**	**SD**	**Mean**	**SD**	***P*** **-value**
Continuous variables							
Age (y)	17.03	0.80	16.09	0.89	17.11	0.68	0.202
Biceps (mm)	12.64	4.16	12.36	4.21	12.92	4.11	0.382
Triceps (mm)	13.42	4.41	13.22	4.80	13.62	4.01	0.252
Subscapular (mm)	23.34	4.38	23.82	4.62	22.86	4.14	0.208
Suprailiac (mm)	21.39	5.17	22.44	5.89	20.34	4.45	0.078
Sum 4 skinfold thickness (mm)^a^	70.79	12.38	71.84	13.57	69.74	11.19	0.439
Body fat (mm)^b^	29.50	3.74	30.20	3.38	28.80	4.09	0.063
Waist circumference (cm)	86.80	10.81	83.78	11.37	89.81	10.25	0.052
Hip circumference (cm)	99.40	9.88	94.94	10.33	103.86	9.43	0.062
Weight (kg)	76.90	12.94	76.77	14.32	77.02	11.55	0.612
Height (cm)	165.01	7.83	164.55	8.19	165.47	7.46	0.475
BMI for age (kg/m^2^)	28.14	3.59	28.23	4.07	28.04	3.11	0.329

*Note.* BMI: Body mass index. ^a^ The sum of the 4 positions refers to the average of biceps, triceps, subscapular, and suprailiac measurements; ^b^ Body fat percentage is the average body fat derived from the equation of Durnin and Womersley (1974) based on the sum of 4 skinfold sites (i.e., biceps, triceps, subscapular, and suprailiac).

 The comparison of outcomes within groups from the intervention to the follow-up visit indicated that the mean body composition (e.g., biceps, triceps, the sum of four skinfold thicknesses, body fat, waist circumference, weight, and BMI for age) and prolonged sedentary behavior in the intervention group decreased significantly (*P* < 0.05)., However, good DB and adequate PA increased significantly (*P* < 0.05). Furthermore, no significant changes were observed in the control group (Table 3).

**Table 3:** Comparison of body composition, dietary behavior, and physical activity data within the intervention and control groups during the participation period (4 weeks), post-participation (8 weeks), and follow-up (12 weeks)

**Outcomes**	**Intervention groups (n=50)**							**Control groups (n=50)**						
	**4 Weeks intervention**		**8 Weeks intervention**		**Follow-up**			**4 Weeks intervention**		**8 Weeks intervention**		**Follow-up**		
**Continuous variables**	**Mean **	**SD**	**Mean **	**SD**	**Mean **	**SD**	***P *** **value**	**Mean **	**SD**	**Mean **	**SD**	**Mean **	**SD**	***P *** **value**
Body composition														
Biceps (mm)	12.26	4.13	12.18	4.03	12.02	4.03	0.020	13.26	4.01	13.84	3.87	13.88	3.96	0.161
Triceps (mm)	13.18	4.75	13.14	4.69	12.96	4.55	0.033	13.60	4.02	14.06	4.02	14.08	4.13	0.094
Subscapular (mm)	23.80	4.54	23.70	4.56	23.02	4.89	0.121	22.88	4.04	23.36	4.29	23.04	4.17	0.070
Suprailiac (mm)	22.42	5.86	22.42	5.86	22.04	6.15	0.075	20.36	4.43	20.48	4.40	20.42	4.44	0.150
Sum 4 skinfold thickness (mm)^a^	71.66	13.46	71.44	13.41	70.04	13.98	0.008	70.10	11.10	71.74	12.01	71.42	11.71	0.091
Body fat (mm)^b^	30.14	3.39	30.13	3.32	29.80	3.42	0.008	28.87	4.08	29.16	4.12	29.11	4.06	0.082
Waist circumference (cm)	83.84	11.38	83.38	11.24	82.26	10.70	0.001	89.79	10.19	90.36	10.37	90.16	10.43	0.281
Hip circumference (cm)	94.94	10.33	94.90	10.31	94.90	10.31	0.395	103.86	9.43	103.86	9.43	103.86	9.43	0.880
Weight (kg)	76.72	14.31	76.34	14.27	76.00	14.06	0.001	77.10	11.79	78.57	11.53	78.33	11.47	0.069
Height (cm)	164.50	8.14	164.50	8.14	164.50	8.14	0.092	165.47	7.46	165.47	7.46	165.47	7.46	0.080
BMI for age (kg/m^2^)	12.26	4.13	12.18	4.03	12.02	4.03	0.020	13.26	4.01	13.84	3.87	13.88	3.96	0.161
Categorical variables	**Number**	**Percent**	**Number**	**Percent**	**Number**	**Percent**	***P *** **value**	**Number**	**Percent**	**Number**	**Percent**	**Number**	**Percent**	***P *** **value**
Dietary behavior							0.001							0.087
Good eating habits	17	34.0	20	40.0	21	42.0		10	20.0	10	20.0	6	12.0	
Moderate eating habits	12	24.0	19	38.0	20	40.0		12	24.0	12	24.0	19	38.0	
Poor eating habits	21	42.0	11	22.0	9	18.0		28	56.0	28	56.0	25	50.0	
Physical activities														
Adequate physical activity	39	78.0	47	94.0	48	96.0	0.001	28	56.0	32	64.0	13	26.0	0.196
Prolonged sedentary behavior	49	98.0	42	84.0	35	70.0	0.001	46	92.0	48	96.0	47	94.0	0.097

*Note.* BMI: Body mass index; SD: Standard deviation. ^a^ The sum of the 4 skinfold sites refers to the average of the biceps, triceps, subscapular, and suprailiac measurements; ^b^ Body fat percentage is the average body fat derived from the equation of Durnin and Womersley (1974) based on the sum of the 4 skinfold sites (i.e., biceps, triceps, subscapular, and suprailiac).

 Additionally, the mean body fat and waist circumference at four weeks post-intervention showed a significant decrease (*P* < 0.05) between the two groups. After eight weeks of intervention, the mean values for biceps, waist circumference, DB, adequate PA, and prolonged sedentary behavior revealed statistical significance (*P* < 0.05). Moreover, at the follow-up visit, the mean differences between the two groups were statistically significant (*P* < 0.05) for biceps, triceps, and waist circumference. Likewise, the proportions of good DB, adequate PA, and prolonged sedentary behavior demonstrated statistical significance (*P* < 0.05), as depicted in Table 4.

**Table 4:** Comparison of body composition, dietary behavior, and physical activity data between the intervention and control groups during the participation period (4 weeks), post-participation (8 weeks), and follow-up (12 weeks)

**Outcomes**	**4 Weeks intervention**				***P*** ** value**	**8 Weeks intervention**				***P *** **value**	**Follow-up **				***P *** **value**
	**Intervention (n=50)**		**Control (n=50)**			**Intervention (n=50)**		**Control (n=50)**			**Intervention (n=50)**		**Control (n=50)**		
**Continuous variables**	**Mean **	**SD**	**Mean **	**SD**		**Mean **	**SD**	**Mean **	**SD**		**Mean **	**SD**	**Mean **	** SD**	
Body composition															
Biceps (mm)	12.26	4.13	13.26	4.01	0.064	12.18	4.03	13.84	3.87	0.003	12.02	4.03	13.88	3.96	0.001
Triceps (mm)	13.18	4.75	13.60	4.02	0.236	13.14	4.69	14.06	4.02	0.059	12.96	4.55	14.08	4.13	0.031
Subscapular (mm)	23.80	4.54	22.88	4.04	0.236	23.70	4.56	23.36	4.29	0.688	23.02	4.89	23.04	4.17	0.978
Suprailiac (mm)	22.42	5.86	20.36	4.43	0.061	22.42	5.86	20.48	4.40	0.072	22.04	6.15	20.42	4.44	0.179
Sum 4 skinfold thickness (mm)^a^	71.66	13.46	70.10	11.10	0.624	71.44	13.41	71.74	12.01	0.817	70.04	13.98	71.42	11.71	0.348
Body fat (mm)	30.14	3.39	28.87	4.08	0.044	30.13	3.32	29.16	4.12	0.144	29.80	3.42	29.11	4.06	0.309
Waist circumference (cm) ^b^	83.84	11.38	89.79	10.19	0.003	83.38	11.24	90.36	10.37	0.001	82.26	10.70	90.16	10.43	0.001
Hip circumference (cm)	94.94	10.33	103.86	9.43	0.062	94.90	10.31	103.86	9.43	0.062	94.90	10.31	103.86	9.43	0.062
Weight (kg)	76.72	14.31	77.10	11.79	0.562	76.34	14.27	78.57	11.53	0.166	76.00	14.06	78.33	11.47	0.182
Height (cm)	164.50	8.14	165.47	7.46	0.475	164.50	8.14	165.47	7.46	0.475	164.50	8.14	165.47	7.46	0.475
BMI for age (kg/m^2^)	28.14	4.01	28.06	3.16	0.702	28.06	4.02	28.61	3.15	0.093	27.94	3.97	28.53	3.13	0.072
Categorical variables	**Number**	**Percent**	**Number**	**Percent**	***P*** **-value**	**Number**	**Percent**	**Number**	**Percent**	***P*** **-value**	**Number**	**Percent**	**Number**	**Percent**	***P*** **-value**
Dietary behavior					0.063					0.007					0.021
Good eating habits	17	34.0	10	20.0		20	40.0	10	20.0		21	42.0	6	12.0	
Moderate eating habits	12	24.0	12	24.0		19	38.0	12	24.0		20	40.0	19	38.0	
Poor eating habits	21	42.0	28	56.0		11	22.0	28	56.0		9	18.0	25	50.0	
Physical Activities															
Adequate physical activity	39	78.0	28	56.0	0.137	47	94.0	32	64.0	0.021	48	96.0	13	26.0	0.030
Prolonged sedentary behavior	49	98.0	46	92.0	0.166	42	84.0	48	96.0	0.029	35	70.0	47	94.0	0.042

*Note.* ^a^ The sum of 4 skinfold sites refers to the average of biceps, triceps, subscapular, and suprailiac; ^b^ Body fat percentage is the average body fat derived from the equation of Durnin and Womersley (1974) based on the sum of 4 skinfold sites (i.e., biceps, triceps, subscapular, and suprailiac).

## Discussion

 The results of this trial showed that the SBM was effective in improving body composition (e.g., biceps, triceps, and waist circumference), consistent with prior research. A previous study conducted an eight-week school-based exercise and a nutrition program led by a nutritionist,^26^ which indicated that combined BM interventions, including diet and PA, are more effective in improving body composition outcomes in overweight adolescents compared to those in the control group. One possible explanation is that SBM focuses on helping students achieve significant changes in their body composition over time by enhancing nutritional knowledge, promoting active behavior, reducing sedentary behavior, and increasing engagement in structured physical activities.^27,28^

 Our results further suggested that SBM is effective in increasing DB, consistent with previous research.^29^ A possible explanation is that components of the SBM, including information on nutrition, appropriate food intake proportions, recommendations for daily food intake, and reducing fast food consumption, along with instructions for recording daily food consumption, could support the students in managing their eating habits and modifying their DB.^30-32^ Some evidence suggests that increasing the nutritional knowledge of individuals can lead to healthier dietary practices such as choosing healthier food, ensuring adequate nutritional intake, and monitoring food consumption, all of which contribute to healthier habits.^33-36^

 Furthermore, SBM significantly increased PA and reduced prolonged sedentary behavior, congruent with prior studies.^37,38^ Promoting PA or reducing sedentary behavior was associated with significant decreases in overweight and body fat percentages and could change health behaviors, leading to healthier habits. One possible explanation is that the “evaluating PA and sedentary behavior” component of the SBM focuses on the participant’s self-monitoring of PA and sedentary behavior. Intentional surveillance and recording of daily physical activities positively influence self-awareness and personal behaviors.^39,40^ Additionally, self-monitoring may help students understand how their daily choices can affect their weight management efforts, highlighting the significant role of PA and reduced sedentary behavior in changing health behaviors.^39^

 This study has some limitations. First, the sample was limited to high school students from two selected colleges in Mae Hong Son province, which may restrict the generalizability of the results to other high school students in other settings. However, it may still reflect health behaviors among overweight individuals within a school-based context. Therefore, further studies should include participants from high schools in different provinces. Second, the SBM was developed for use in a school-based context, specifically in overweight high school students, who may have different experiences with food consumption habits and PA compared to those seeking treatment for overweight or obesity in clinical settings. Therefore, caution should be exercised when generalizing the results to other populations. Despite these limitations, our study demonstrated that the SBM is a feasible intervention that can improve body composition, DB, and PA while reducing sedentary behavior among overweight high school students. Therefore, SBM has the potential to effectively modify students’ health behaviors.

**Highlights**

- A school-based behavioral modification (SBM) program is effective in improving physical activity (PA) and dietary behaviors (DBs) among high school students with overweight.
- The SBM program could reduce sedentary behavior and change body composition.
- Social cognitive theory-based health education is effective for improving health behavior outcomes.

## Conclusion

 The SBM program effectively improved students’ PA, DB, and body composition compared to the control group, confirming that the program is effective. These findings suggest that the SBM should be implemented to promote healthy behavior among overweight high school students. Future studies should be conducted in different areas. Additionally, integrating the program into health education classes and school curricula can further amplify its impact and can be applied in related research in different contexts and situations.

## Acknowledgements

 We are grateful to the Faculty of Public Health, Mahasarakham University, for their financial support. We would also like to sincerely thank all subjects for their participation in the study.

 Thidarat Somdee (PhD)^1^, Supattarayan Thongjit (PhD)^2^, and Suneerat Yangyuen (PhD)^1^*

## Competing Interests

 The authors declare no conflict of interests.

## Ethical Approval

 This study was approved by the Research Ethics Committee at Mahasarakham University (certification numbers 337-280/2564, October 21, 2021, and 102-128/2566, March 23, 2023). Furthermore, it was registered with the Clinical Trials Registry of Thailand (TCTR) under the auspices of the Medical Research Foundation (MRF), under the registration number TCTR20220606004 on June 6, 2022.

## Funding

 This research was financially supported by the Faculty of Public Health, Mahasarakham University.
